# Time-Dependent Changes in NLR, PLR, SII, and SIRI During Intraoperative Cardiopulmonary Bypass in CABG Patients and Their Association with In-Hospital Mortality

**DOI:** 10.3390/jcm15145351

**Published:** 2026-07-08

**Authors:** Burak Toprak, Abdulkadir Bilgiç, Rahime Akın, Mustafa Ekici, Ahmet Turhan Kılıç, Özkan Karaca, Nihat Söylemez, Sonay Oğuz, Mehmet Ballı, Mahmut Yılmaz, Ali Orçun Sürmeli, Serdar Keçeoğlu

**Affiliations:** 1 Department of Cardiovascular Surgery, Mersin City Education and Research Hospital, 33240 Mersin, Turkey; ahmetturhank@gmail.com (A.T.K.); sonayoguz1973@gmail.com (S.O.); 2 Department of Cardiovascular Surgery, Mersin University Faculty of Medicine Hospital, 33110 Mersin, Turkey; bilgicabdulkadir@gmail.com (A.B.); rahime_akn@hotmail.com (R.A.); 3 Department of Emergency Medicine, Mersin Provincial Health Directorate, 33060 Mersin, Turkey; dr.mustafaekici@hotmail.com; 4 Department of Cardiology, Mersin City Education and Research Hospital, 33240 Mersin, Turkey; md.ozkrc@gmail.com (Ö.K.); drnihatsylmz@gmail.com (N.S.); dr_mehmetballi@hotmail.com (M.B.); myilmaz510@hotmail.com (M.Y.); orcun_surmeli@hotmail.com (A.O.S.); serdarkeceoglu@gmail.com (S.K.)

**Keywords:** coronary artery bypass grafting, cardiopulmonary bypass, systemic immune-inflammation index, systemic inflammatory response index, neutrophil-to-lymphocyte ratio, platelet-to-lymphocyte ratio, mortality prediction, perioperative risk stratification

## Abstract

**Background:** Systemic inflammation plays a central role in determining postoperative outcomes in patients undergoing isolated coronary artery bypass grafting with cardiopulmonary bypass. Traditional inflammatory indices such as the neutrophil-to-lymphocyte ratio and the platelet-to-lymphocyte ratio have prognostic value; however, their dynamic behavior during cardiopulmonary bypass remains insufficiently characterized. More comprehensive indices, including the systemic immune-inflammation index and the systemic inflammatory response index, may help characterize early intraoperative inflammatory activity; however, their prognostic relevance should be regarded as exploratory and requires prospective validation. **Methods:** This retrospective nested case–control study included 245 patients who underwent isolated coronary artery bypass grafting, and intraoperative inflammatory indices during cardiopulmonary bypass were evaluated. Because of the nested case–control design, mortality cases were intentionally overrepresented to improve statistical power; therefore, the observed mortality rate does not reflect the true institutional mortality rate. Inflammatory indices (NLR, PLR, SII, and SIRI) were calculated at induction, at the 5th, 45th, and 90th minutes during cardiopulmonary bypass, and in the early postoperative period. Associations between these indices and in-hospital mortality were evaluated using univariate and multivariable logistic regression analyses. Predictive performance was assessed using receiver operating characteristic (ROC) curve analysis and the area under the curve (AUC). **Results:** The final enriched analytical sample consisted of 51 mortality cases and 194 randomly sampled surviving controls. During cardiopulmonary bypass, inflammatory indices, particularly at the 5th minute, were significantly higher in patients who experienced mortality (*p* < 0.001 for all major indices). SII demonstrated the strongest predictive performance at the 5th minute (AUC = 0.790), followed by SIRI (AUC = 0.765), PLR (AUC = 0.687), and NLR (AUC = 0.681). In multivariable analysis, SII and SIRI measured at the 5th minute remained independent predictors of mortality. The addition of 5th-minute SII to the limited study-specific clinical model, which included age, ejection fraction, and preoperative creatinine, improved exploratory discrimination for in-hospital mortality (with AUC increasing from 0.698 to 0.797). **Conclusions:** Early intraoperative assessment of inflammatory indices during cardiopulmonary bypass may provide additional prognostic information in patients undergoing coronary artery bypass grafting. Composite indices, particularly SII and SIRI, showed stronger exploratory discrimination than traditional inflammatory markers in this enriched analytical sample. However, these findings should be considered hypothesis-generating and require prospective external validation before use in perioperative risk stratification or clinical decision-making can be recommended.

## 1. Introduction

Perioperative major adverse events represent one of the leading causes of both morbidity and mortality in surgical patients, clearly emphasizing the critical importance of effective risk stratification for improving clinical outcomes [1]. Therefore, the use of inflammatory biomarkers in perioperative risk assessment has been increasing, playing a significant role in optimizing patient management [2]. In particular, white blood cell subtypes constitute the fundamental components of the inflammatory response and play a central role in the systemic inflammatory response and immune regulation. Hematological indices derived from complete blood count parameters, including the neutrophil-to-lymphocyte ratio (NLR) and the platelet-to-lymphocyte ratio (PLR), are widely used for the assessment of systemic inflammation and thromboinflammatory activity [3]. NLR reflects the balance between inflammatory and immune responses, whereas PLR incorporates both platelet activation and inflammation [3,4]. More recently, composite indices such as the systemic immune-inflammation index (SII) and systemic inflammatory response index (SIRI) have been proposed to provide a broader representation of inflammatory activity by integrating multiple cellular components. Because all of these indices are calculated from routinely available laboratory parameters, they may offer practical tools for perioperative inflammatory assessment during cardiopulmonary bypass. Therefore, a comparative evaluation of traditional and composite inflammatory markers may provide additional insight into their relative prognostic utility.

However, the inflammatory response that develops during cardiopulmonary bypass (CPB) is not limited to alterations in the neutrophil–lymphocyte balance alone. The CPB-associated inflammation represents a complex and multidimensional immune response characterized by neutrophil activation, monocyte-mediated cytokine release, platelet activation, endothelial dysfunction, and the simultaneous activation of the coagulation cascade [5]. Therefore, evaluating the inflammatory process based solely on two cell subtypes may not fully reflect the multilayered immune response triggered by CPB. In this context, the systemic immune-inflammation index and the systemic inflammatory response index have emerged as promising composite biomarkers that may provide a more comprehensive representation of inflammatory activity. SII is calculated by dividing the product of neutrophil and platelet counts by the lymphocyte count and integrates inflammation (neutrophils), thrombotic activity (platelets), and immune regulation (lymphocytes) into a single parameter [6]. During cardiopulmonary bypass, contact between blood and artificial surfaces increases platelet activation, while neutrophil degranulation and the release of reactive oxygen species further exacerbate endothelial injury. Concurrent lymphocyte suppression indicates impaired immune regulation [7]. Therefore, SII may provide a more comprehensive representation of the inflammatory and thromboinflammatory processes associated with CPB by integrating neutrophil, platelet, and lymphocyte components into a single index.

Similarly, SIRI is calculated by dividing the product of neutrophil and monocyte counts by the lymphocyte count and is particularly important because it incorporates the monocyte-mediated cytokine response into the equation [8]. During CPB, monocytes play a key role in the production of interleukin-6, tumor necrosis factor-alpha, and other proinflammatory mediators and occupy a central position in the development of systemic inflammatory response syndrome (SIRS) [7]. The inflammatory activity of monocytes is a major determinant of increased endothelial permeability, microvascular dysfunction, and organ injury [9]. Therefore, SIRI reflects not only the acute neutrophil response but also the sustained inflammatory burden mediated by monocytes.

Elective isolated coronary artery bypass grafting performed under general anesthesia, particularly when cardiopulmonary bypass is used, triggers a significantly more pronounced systemic inflammatory response compared with other surgical procedures due to the additional physiological stress imposed by extracorporeal circulation [10]. This inflammatory response is further intensified by multiple factors, including blood contact with artificial surfaces of the bypass circuit, ischemia–reperfusion injury, endotoxemia, surgical trauma, and hemodynamic fluctuations. Consequently, serious postoperative complications such as myocardial dysfunction, respiratory failure, acute kidney injury, neurological complications, coagulation disorders, and hepatic dysfunction may develop, potentially resulting in multi-organ failure in severe cases [11].

In the current literature, the prognostic value of inflammatory indices has predominantly been evaluated at a single preoperative or postoperative time point, and studies investigating the dynamic changes in the inflammatory response during CPB remain limited. However, the inflammatory response during CPB may initially exhibit a marked elevation in the early phase, followed by immune suppression or immune paralysis in later stages. Considering this biphasic response model, evaluating inflammatory indices not only through static measurements but also through their temporal changes may provide greater clinical relevance.

Given the strong association between perioperative inflammation and postoperative mortality, the dynamic and comparative evaluation of inflammatory indices may provide a significant advantage in surgical risk stratification. Although several previous studies have evaluated inflammatory biomarkers in patients undergoing cardiac surgery, most have focused on a single marker measured at either a preoperative or postoperative time point. Data regarding the dynamic behavior of multiple inflammatory indices during cardiopulmonary bypass remain scarce. Furthermore, direct comparisons between traditional indices such as NLR and PLR and more comprehensive composite indices such as SII and SIRI within the same CABG cohort are limited. The present study differs from previous investigations by simultaneously evaluating NLR, PLR, SII, and SIRI at multiple intraoperative cardiopulmonary bypass time points and by comparing their relative prognostic performance for in-hospital mortality. This approach allows a more comprehensive assessment of the temporal inflammatory response during cardiopulmonary bypass. Accordingly, the present study evaluated the temporal changes in NLR, PLR, SII, and SIRI during cardiopulmonary bypass and the postoperative period and compared their performance for predicting in-hospital mortality in patients undergoing isolated CABG. This study also aimed to determine the exploratory incremental contribution of these indices to mortality prediction when combined with a limited study-specific clinical model and to explore their potential utility for early intraoperative risk assessment during cardiopulmonary bypass.

## 2. Materials and Methods

### 2.1. Study Design and Ethical Considerations

This retrospective single-center nested case–control study was conducted at the Department of Cardiovascular Surgery, Mersin University Faculty of Medicine, and included patients who underwent isolated CABG using cardiopulmonary bypass between 1 May 2018, and 1 March 2023. During the study period, a total of 960 CABG procedures were screened from the institutional surgical database. Of these, 57 urgent or emergency CABG procedures and 29 redo CABG procedures were excluded according to the predefined eligibility criteria. No off-pump CABG or combined surgical procedures were present in this screened population. After applying these criteria, 874 adult patients who underwent elective isolated on-pump CABG constituted the eligible source population. Among this eligible source population, 51 patients experienced in-hospital mortality, and all mortality cases were included in the case group. The remaining 823 patients survived to hospital discharge and constituted the eligible survivor pool. From this survivor pool, 202 patients were initially selected by simple random sampling as potential controls. After exclusion of 8 patients because of incomplete serial inflammatory measurements or missing key laboratory/mortality data, 194 surviving controls remained eligible for analysis. Therefore, the final enriched nested case–control analytical sample consisted of 245 patients, including 51 mortality cases and 194 randomly sampled surviving controls. In this nested case–control framework, mortality cases were intentionally overrepresented to improve statistical power for biomarker analyses; consequently, the observed mortality proportion in the analytical sample should not be interpreted as the actual institutional mortality rate. Consequently, the observed mortality proportion should not be interpreted as the actual institutional mortality rate. This study was conducted and reported in accordance with the Reporting of studies Conducted using Observational Routinely collected health Data (RECORD) statement, an extension of the STROBE guidelines. The inclusion criteria were as follows: adult patients aged ≥18 years who underwent elective isolated coronary artery bypass grafting using cardiopulmonary bypass during the predefined study period, availability of complete intraoperative cardiopulmonary bypass blood count measurements at all predefined time points, and availability of complete in-hospital mortality data. To preserve procedural and hemodynamic homogeneity, patient selection was performed meticulously, and only patients with standardized operative and perioperative data were included in the final analysis. The exclusion criteria were as follows:

- Urgent or emergency surgery
- Valve surgery or combined CABG and valve surgery
- Aortic surgery
- Congenital cardiac surgery
- Redo cardiac surgery
- Minimally invasive procedures
- Off-pump CABG
- Pediatric cases
- Active preoperative infection
- Missing key laboratory or mortality data
- Preoperative extracorporeal membrane oxygenation support
- Salvage surgery during active cardiogenic shock
- Ongoing cardiopulmonary resuscitation before surgery
- Major perioperative data inconsistency or incomplete serial inflammatory measurements

### 2.2. Data Collection and Inflammatory Indices

Demographic characteristics, clinical comorbidities, surgical data, and laboratory parameters of all patients were retrospectively obtained from the hospital electronic medical record system. To improve retrospective data reliability, all laboratory and operative variables were independently cross-checked using both institutional electronic medical records and archived perioperative anesthesia/perfusion records when available. The analyzed clinical variables included age, sex, the presence of diabetes mellitus, the presence of hypertension, ejection fraction, cardiopulmonary bypass duration, and aortic cross-clamp time. The study population consisted exclusively of adult patients undergoing isolated coronary artery bypass grafting with cardiopulmonary bypass. Pediatric cases were excluded. Only elective isolated CABG procedures were included to preserve procedural and hemodynamic homogeneity, whereas redo procedures, urgent operations, and minimally invasive procedures were excluded. Patients requiring preoperative extracorporeal membrane oxygenation support, salvage procedures during active cardiogenic shock, or ongoing cardiopulmonary resuscitation were excluded from the study because these extreme hemodynamic conditions could substantially alter inflammatory responses independently of the cardiopulmonary bypass process. Patients with active preoperative infection, defined by documented infectious diagnosis, overt clinical signs of infection, or markedly elevated inflammatory markers before surgery, were excluded from the analysis. All surgical procedures were performed using standardized cardiopulmonary bypass protocols. Non-pulsatile flow was maintained with a target pump flow of 2.2–2.5 L/min/m^2^, and systemic temperature management was performed using mild-to-moderate hypothermia according to intraoperative clinical requirements. Hematocrit levels during cardiopulmonary bypass were maintained according to institutional perfusion protocols. Myocardial protection was achieved using standardized cardioplegia strategies administered by the operating surgical team. Cardiopulmonary bypass duration and aortic cross-clamp times were recorded for all patients and included in the operative dataset. Hematological parameters were measured using an automated hematology analyzer as part of routine clinical practice. Complete blood count measurements were obtained at predefined perioperative time points to allow a dynamic evaluation of inflammatory response kinetics, with a particular focus on intraoperative cardiopulmonary bypass measurements: the preoperative baseline period (before induction), the 5th minute, 45th minute, and 90th minute during cardiopulmonary bypass, and the early postoperative period. The 90 min measurement was obtained only from patients who remained on active cardiopulmonary bypass at the predefined 90 min sampling interval. Patients whose cardiopulmonary bypass duration was shorter than 90 min did not contribute data to the 90 min analyses. Accordingly, all analyses involving the 90 min time point were restricted to patients who were still undergoing cardiopulmonary bypass at that time. The baseline measurement (time zero) was defined as the last complete blood count obtained immediately before anesthesia induction. The postoperative measurement was defined as the complete blood count obtained at the 24th postoperative hour, which was selected as a standardized early postoperative time point to reduce the confounding effects of immediate perioperative hemodilution and acute transfusion-related fluctuations. Patients who died before the 24th postoperative hour and therefore did not have an available 24 h postoperative complete blood count were not included in the postoperative inflammatory index analyses because of the complete-case analytical approach. Therefore, the postoperative inflammatory index findings should be interpreted as applying to patients with available 24 h postoperative laboratory measurements and may be influenced by survivor bias. Using the obtained neutrophil, lymphocyte, platelet, and monocyte values, the following inflammatory indices were calculated: NLR, PLR, systemic immune-inflammation index (SII; platelet × neutrophil/lymphocyte), and systemic inflammatory response index (SIRI; neutrophil × monocyte/lymphocyte). These indices were calculated separately for all time points, and their associations with mortality were analyzed.

### 2.3. Outcome Definition

The primary endpoint was defined as all-cause in-hospital mortality occurring during the index hospitalization period following CABG surgery. A cause-specific mortality classification was not systematically available for all patients because of the retrospective design of the study. Therefore, mortality was evaluated as all-cause in-hospital mortality rather than specific cardiac, infectious, or surgical mortality subtypes. Since postoperative mortality after CABG is frequently multifactorial and may involve hemodynamic deterioration, low cardiac output syndrome, infection, multiorgan dysfunction, or simultaneous procedure-related complications, a unified all-cause mortality endpoint was preferred to reduce classification bias. No predefined secondary endpoints were included because the primary objective of the study was focused specifically on the prognostic association between dynamic inflammatory indices and in-hospital mortality.

### 2.4. Statistical Analysis

All statistical analyses were performed to compare clinical, biochemical, and inflammatory parameters between patients who experienced mortality (Ex) and those who did not (Non-Ex), and to evaluate the effects of these variables on in-hospital mortality.

First, the distribution characteristics of continuous variables were assessed using the Kolmogorov–Smirnov test. Continuous variables with a normal distribution were expressed as mean ± standard deviation (mean ± SD), and comparisons between two independent groups were performed using the independent samples Student’s *t*-test. Continuous variables without a normal distribution were presented as median (minimum–maximum), and comparisons between groups were performed using the Mann–Whitney U test. Categorical variables were expressed as number (*n*) and percentage (%), and comparisons were performed using the Pearson Chi-square test or Fisher’s exact test when expected cell counts were low.

Univariate logistic regression analysis was performed to evaluate clinical and operative variables associated with mortality. In this analysis, the odds ratio (OR), 95% confidence interval (confidence interval, CI), and *p*-value calculated using the Wald test were reported for each variable. Continuous variables were included in the model as continuous variables, and no categorical cut-off values were used.

To evaluate the time-dependent effects of inflammatory indices (NLR, PLR, SII, and SIRI) on mortality, separate univariate logistic regression analyses were performed for each index at all measurement time points (induction, the 5th, 45th, and 90th minutes, and the postoperative period). Although the dataset included repeated measurements obtained from the same patients, each time point was analyzed separately to evaluate time-specific prognostic performance and clinically actionable perioperative decision windows rather than longitudinal within-subject trajectories. This approach may introduce residual within-subject correlation and should therefore be interpreted as time point-specific predictive modeling rather than a formal repeated-measures analysis. Mixed-effects models and generalized estimating equations (GEE) were not selected because the primary objective was not to characterize longitudinal inflammatory trajectories or average within-subject changes over time. Instead, the study was designed to assess the prognostic value of inflammatory indices at predefined clinically relevant perioperative time points. Consequently, each measurement point was evaluated as an independent prognostic assessment, although the potential influence of within-subject correlation remains a recognized limitation. Therefore, the findings derived from separate time point-specific models should not be interpreted as statistically independent longitudinal effects. Because inflammatory indices were repeatedly measured in the same individuals, residual dependency among serial observations may have influenced variance estimates, confidence intervals, and the apparent strength of some associations. Accordingly, these analyses should be regarded as exploratory time-specific predictive assessments rather than definitive repeated-measures inference. To further address the repeated-measurement structure and to reduce the potential influence of baseline differences, additional exploratory baseline-adjusted analyses were performed. For each inflammatory index, the absolute change from baseline was calculated as the value at each cardiopulmonary bypass time point minus the induction value. The percentage change from baseline was calculated as [(cardiopulmonary bypass time point value − induction value)/induction value] × 100. These analyses were performed for the 5th, 45th, and 90th minute measurements, with the 90 min analyses restricted to patients who remained on cardiopulmonary bypass for ≥90 min. In these analyses, the indices were included in the model as continuous variables, and the effect of each unit increase on mortality was expressed as OR and 95% CI.

Multivariable logistic regression models were constructed to determine the additional prognostic contribution of inflammatory indices beyond a limited study-specific clinical model. The clinical base model was structured to include age, ejection fraction, and preoperative creatinine levels. Established operative risk scores such as STS and EuroSCORE II were not consistently available in the retrospective dataset and therefore could not be incorporated into the adjustment models. Therefore, any observed improvement in discrimination after adding inflammatory indices should be interpreted as incremental value beyond this limited study-specific model, rather than beyond contemporary standard surgical risk assessment. This should be considered an important limitation, as these scores may capture perioperative risk dimensions beyond the inflammatory burden alone. Additional perioperative laboratory variables such as sodium, liver enzymes, hemoglobin, and postoperative creatinine were not included in the multivariable models, as these parameters may reflect downstream consequences of perioperative complications rather than baseline confounders, and their inclusion could introduce overadjustment bias. Similarly, cardiopulmonary bypass duration and cross-clamp time, although significant in the univariate analysis, were not included in the primary multivariable base model because they are highly procedure-dependent variables that may partially mediate the relationship between inflammatory activation and mortality rather than function as independent baseline confounders. To assess the robustness of the primary findings to operative exposure variables, predefined exploratory sensitivity models were constructed by adding cardiopulmonary bypass duration and aortic cross-clamp time to the primary adjustment models. These analyses were performed only as sensitivity analyses and were not considered part of the primary adjustment strategy. Hemodilution-related variables, intraoperative hematocrit trajectories, circuit priming volume, and perioperative transfusion burden were also considered as potential confounders. However, complete and consistently structured data for these variables were not available for all patients in the retrospective dataset. Therefore, they could not be incorporated into the primary multivariable or sensitivity models and were addressed as residual confounding factors. Subsequently, separate models were created by adding 5th-minute NLR, 5th-minute SII, and 5th-minute SIRI to the clinical base model. In addition, models including combinations of inflammatory indices (NLR + SII, NLR + SIRI, and SII + SIRI) were analyzed to evaluate their combined effects. PLR was intentionally not included in the combined multivariable models because its biological components substantially overlap with SII and partially with NLR, which could increase redundancy without providing substantial incremental information. Model selection was based on clinical relevance and variables found to be statistically significant in univariate analysis. The number of predictors entered into each multivariable model was additionally restricted according to the events-per-variable principle, maintaining an EPV ratio greater than 10 to reduce overfitting risk. The potential for multicollinearity was assessed using the variance inflation factor (VIF) and correlation coefficients. Models containing a single inflammatory index were prespecified as the primary analyses, whereas combination models including multiple correlated inflammatory indices were considered secondary exploratory analyses and interpreted with caution when VIF values exceeded acceptable thresholds.

The discriminative performance of the models was evaluated using receiver operating characteristic (ROC) curve analysis, and the area under the curve (AUC) was calculated for each model. AUC values were compared to assess the ability of the models to discriminate mortality. Additionally, separate ROC analyses were performed for each inflammatory index at each time point, and optimal cut-off values were determined using the Youden index. Sensitivity and specificity were calculated for each cut-off value. For ROC analyses, mortality was coded as the positive outcome. For postoperative inflammatory indices, lower values were associated with mortality; therefore, ROC analyses for the postoperative NLR, PLR, SII, and SIRI were direction-adjusted by using the inverse marker direction so that AUC values reflected discriminatory ability rather than the raw direction of association. Accordingly, postoperative AUC values greater than 0.5 indicate that lower postoperative inflammatory index values were associated with mortality. Because the nested case–control design intentionally enriched mortality cases, the calibration metrics, absolute risk estimates, predictive values, and Youden-derived cut-off values were interpreted as exploratory. These measures should not be directly extrapolated to an unselected consecutive CABG population without external validation.

Statistical significance was evaluated using a two-tailed approach, and a *p*-value < 0.05 was considered statistically significant. The primary analytical hypothesis was defined as the association between the systemic immune-inflammation index measured at the 5th minute of cardiopulmonary bypass and in-hospital mortality, together with its exploratory incremental discriminatory contribution when added to the limited study-specific clinical model. Analyses involving other inflammatory indices, additional time points, postoperative measurements, combined inflammatory index models, ROC-derived cut-off values, and baseline-adjusted change analyses were considered secondary exploratory analyses. Because the study was designed as an exploratory and hypothesis-generating investigation with a predefined primary analytical focus rather than as a confirmatory multiple-hypothesis testing study, a formal false-discovery-rate correction was not applied to all secondary comparisons. We acknowledge that this approach may increase the risk of a type I error, particularly for the secondary analyses involving multiple indices, time points, and model comparisons. Therefore, isolated statistically significant findings should be interpreted cautiously. Greater emphasis was placed on consistency across time points, biological plausibility, effect size direction, and prespecified model performance rather than on single *p*-values alone. These findings should be validated in prospective cohorts with predefined correction strategies for multiple testing.

#### Software

All statistical analyses were performed using Python (version 3.12). Data processing and cleaning were conducted using the pandas and numpy libraries, statistical analyses were performed with scipy and statsmodels, and ROC analysis and classification performance assessments were carried out using the scikit-learn package. The analysis workflow was conducted in accordance with the principles of reproducibility. A post hoc statistical power analysis was additionally performed based on the primary discriminative performance of the 5th-minute SII model for in-hospital mortality. Using the observed event proportion, sample size, and ROC-derived AUC, the achieved statistical power was calculated to assess the adequacy of the study cohort. Furthermore, internal validation of the multivariable prediction models was performed using bootstrap resampling (1000 iterations) to estimate optimism-corrected AUC values and reduce the risk of model overfitting. In addition to the discrimination analysis, the calibration performance of the primary multivariable models was assessed using calibration slope/intercept evaluation and the Hosmer–Lemeshow goodness-of-fit approach. Model calibration findings were interpreted together with the discrimination metrics to provide a more comprehensive evaluation of predictive performance.

## 3. Results

During the study period, 960 CABG procedures were screened from the institutional surgical database. After the exclusion of 57 urgent or emergency CABG procedures and 29 redo CABG procedures, 874 adult patients who underwent elective isolated on-pump CABG constituted the eligible source population. No off-pump CABG or combined surgical procedures were present in the screened population. A total of 51 patients experienced in-hospital mortality, and all mortality cases were included in the case group. Among the remaining 823 survivors, 202 patients were randomly selected as potential controls. After the exclusion of eight patients because of incomplete serial inflammatory measurements or missing key laboratory/mortality data, 194 surviving controls remained eligible for analysis. Therefore, the final enriched nested case–control analytical sample included 245 patients, consisting of 51 in-hospital mortality cases and 194 randomly sampled surviving controls. Because the mortality cases were intentionally enriched within the nested case–control design, the proportion of mortality cases in the analytical sample should not be interpreted as the institutional or cohort-level mortality rate. Patients were analyzed according to mortality status as mortality cases (Ex) and sampled surviving controls (Non-Ex). To enable dynamic evaluation of the inflammatory response, NLR, PLR, SII, and SIRI values were measured and compared during the preoperative period, at the 5th, 45th, and 90th minutes during cardiopulmonary bypass, and in the early postoperative period. A total of 184 patients (75.1% of the study cohort) remained on cardiopulmonary bypass at the 90 min time point and were therefore included in the analyses involving 90 min inflammatory index measurements.

In addition, the discriminative performance of each index in predicting mortality was analyzed using receiver operating characteristic (ROC) curve analysis and comparatively evaluated.

The baseline characteristics of the study population are presented in Table 1. The mean age of the patients was 59.3 ± 12.5 years, with a median age of 61 years and a range from 19 to 80 years. The cohort consisted of 156 male patients (63.7%) and 89 female patients (36.3%). The enriched analytical sample consisted of 51 mortality cases and 194 sampled surviving controls. Diabetes mellitus was present in 125 patients (51.0%), and hypertension was present in 147 patients (60.0%). The mean cardiopulmonary bypass pump time was 117.2 ± 44.3 min, with a median of 110 min and a range of 36 to 254 min. The mean cross-clamp time was 69.1 ± 29.1 min, with a median of 62 min and a range from 12 to 143 min (Table 1).

The comparison of laboratory parameters between mortality and survival groups is presented in Table 2. Postoperative sodium levels were significantly lower in patients who experienced mortality compared with survivors (136.63 ± 2.64 vs. 138.07 ± 2.80, *p* < 0.001). Postoperative ALT levels were significantly higher in the mortality group (29.33 ± 15.41 vs. 24.86 ± 12.71, *p* = 0.038). Postoperative AST levels were also significantly elevated in patients who experienced mortality (47.57 ± 20.53 vs. 35.96 ± 16.34, *p* < 0.001). Postoperative CRP levels were significantly higher in the mortality group compared to survivors (84.28 ± 26.69 vs. 66.86 ± 23.64, *p* < 0.001). Postoperative hemoglobin levels were significantly lower in patients who experienced mortality (9.77 ± 1.61 vs. 10.52 ± 1.45, *p* = 0.002). Postoperative white blood cell counts were significantly higher in the mortality group (14.92 ± 3.91 vs. 12.41 ± 3.27, *p* < 0.001). Postoperative creatinine levels were significantly elevated in patients with mortality compared with survivors (1.98 ± 1.07 vs. 1.39 ± 0.70, *p* < 0.001). Preoperative lymphocyte counts were significantly lower in patients who experienced mortality (1.76 ± 0.57 vs. 2.06 ± 0.64, *p* = 0.002). Postoperative neutrophil counts were significantly higher in the mortality group (12.40 ± 3.44 vs. 10.10 ± 2.97, *p* < 0.001). Postoperative lymphocyte counts were significantly lower in patients who experienced mortality (1.08 ± 0.40 vs. 1.42 ± 0.51, *p* < 0.001). Postoperative platelet counts were significantly lower in the mortality group (171.51 ± 47.92 vs. 206.59 ± 50.97, *p* < 0.001). Postoperative monocyte counts were significantly higher in patients who experienced mortality (0.86 ± 0.28 vs. 0.67 ± 0.23, *p* < 0.001) (Table 2).

The comparison of dynamically changing inflammatory indices during cardiopulmonary bypass between mortality and survival groups is presented in Table 3. Baseline PLR and SII values were significantly higher in patients who experienced mortality compared with survivors (PLR ind: 228.26 ± 132.23 vs. 154.70 ± 76.82, *p* < 0.001; SII ind: 1010.50 ± 411.62 vs. 776.56 ± 594.64, *p* = 0.001). At the 5th minute during cardiopulmonary bypass time point, NLR, PLR, SII, and SIRI values were significantly higher in the mortality group compared with the survival group (NLR 5: 2.84 ± 0.99 vs. 2.17 ± 1.19, *p* < 0.001; PLR 5: 218.94 ± 116.78 vs. 140.49 ± 64.76, *p* < 0.001; SII 5: 407.00 ± 132.64 vs. 258.17 ± 180.62, *p* < 0.001; SIRI 5: 3.40 ± 1.64 vs. 2.03 ± 1.33, *p* < 0.001). At the 45th minute, PLR, SII, and SIRI values were significantly higher in patients who experienced mortality (PLR 45: 217.47 ± 101.23 vs. 166.32 ± 69.09, *p* < 0.001; SII 45: 585.50 ± 278.05 vs. 388.64 ± 212.34, *p* < 0.001; SIRI 45: 2.86 ± 1.27 vs. 2.06 ± 1.06, *p* < 0.001). At the 90th minute during cardiopulmonary bypass measurement, PLR, SII, and SIRI values were also significantly higher in the mortality group (PLR 90: 220.77 ± 93.59 vs. 189.60 ± 74.54, *p* = 0.020; SII 90: 655.78 ± 330.90 vs. 489.05 ± 272.72, *p* < 0.001; SIRI 90: 2.72 ± 1.18 vs. 2.15 ± 1.08, *p* = 0.003). In contrast, postoperative NLR, PLR, SII, and SIRI values were significantly lower in the mortality group compared with survivors (NLR post: 8.27 ± 4.86 vs. 14.87 ± 9.09, *p* < 0.001; PLR post: 127.64 ± 92.97 vs. 261.33 ± 161.58, *p* < 0.001; SII post: 1356.57 ± 731.41 vs. 2473.81 ± 1676.08, *p* < 0.001; SIRI post: 8.63 ± 5.61 vs. 12.70 ± 8.64, *p* < 0.001) (Table 3).

**Figure 1:**
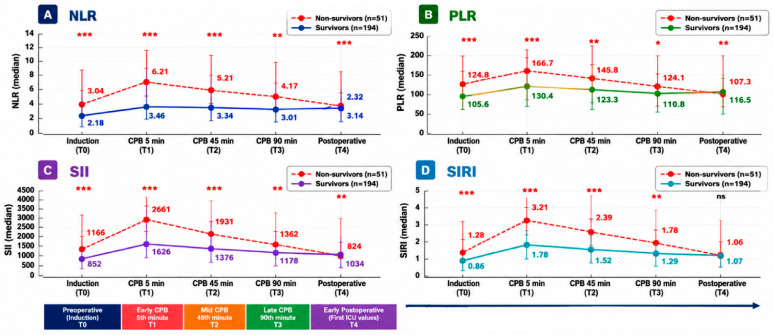
Temporal changes in inflammatory indices during cardiopulmonary bypass according to in-hospital mortality status. Temporal profiles of neutrophil-to-lymphocyte ratio (NLR, panel (**A**)), platelet-to-lymphocyte ratio (PLR, panel (**B**)), systemic immune-inflammation index (SII, panel (**C**)), and systemic inflammatory response index (SIRI, panel (**D**)) measured at induction, the 5th, 45th, and 90th minutes of cardiopulmonary bypass, and the early postoperative period in patients with and without in-hospital mortality. Values are presented as mean ± standard deviation. Patients who experienced in-hospital mortality demonstrated markedly higher inflammatory index values during cardiopulmonary bypass, particularly at the 5th minute, whereas postoperative values were lower compared with survivors. NLR = neutrophil-to-lymphocyte ratio; PLR = platelet-to-lymphocyte ratio; SII = systemic immune-inflammation index; SIRI = systemic inflammatory response index; CPB = cardiopulmonary bypass. * *p* < 0.05, ** *p* < 0.01, *** *p* < 0.001, ns: not significant (Mann-Whitney U test).

The temporal evolution of inflammatory indices according to mortality status is illustrated in Figure 1. All indices demonstrated a pronounced increase during the early phase of cardiopulmonary bypass, with the greatest between-group separation observed at the 5th minute. Although inflammatory marker levels gradually declined thereafter, patients who experienced mortality maintained higher intraoperative values throughout cardiopulmonary bypass. In contrast, the postoperative NLR, PLR, SII, and SIRI values were lower in the mortality group than in survivors, demonstrating a reversal of the intraoperative pattern (Figure 1).

Baseline-adjusted absolute and percentage change analyses are presented in Table 4. At the 5th minute of cardiopulmonary bypass, SIRI showed a significantly greater absolute and percentage increase from baseline in mortality cases compared with the sampled surviving controls. In addition, the percentage reduction in SII from baseline was less pronounced in the mortality cases than in the sampled surviving controls. However, not all raw between-group differences persisted after the baseline adjustment, indicating that early cardiopulmonary bypass inflammatory measurements may reflect both the baseline inflammatory burden and perioperative factors such as hemodilution, circuit priming, and hematologic redistribution. Therefore, these baseline-adjusted analyses support a more cautious interpretation of the dynamic inflammatory response during cardiopulmonary bypass (Table 4).

The univariate logistic regression analysis for in-hospital mortality is presented in Table 5. Age was significantly associated with an increased mortality risk (OR: 1.062, 95% CI: 1.028–1.097, *p* < 0.001). Pump time was also significantly associated with mortality (OR: 1.043, 95% CI: 1.030–1.055, *p* < 0.001). Similarly, cross-clamp time was significantly associated with increased mortality risk (OR: 1.115, 95% CI: 1.081–1.150, *p* < 0.001) (Table 5).

The univariate logistic regression analysis of the inflammatory indices is presented in Table 5. Baseline PLR and SII were significantly associated with mortality (PLR ind: OR: 1.007, 95% CI: 1.004–1.010, *p* < 0.001; SII ind: OR: 1.001, 95% CI: 1.000–1.001, *p* = 0.016). At the 5 min CPB measurement, NLR, PLR, SII, and SIRI were significantly associated with mortality (NLR 5: OR: 1.583, 95% CI: 1.219–2.056, *p* < 0.001; PLR 5: OR: 1.010, 95% CI: 1.006–1.014, *p* < 0.001; SII 5: OR: 1.004, 95% CI: 1.003–1.006, *p* < 0.001; SIRI 5: OR: 1.742, 95% CI: 1.408–2.155, *p* < 0.001). At the 45th minute, PLR, SII, and SIRI were significantly associated with mortality (PLR 45: OR: 1.003, 95% CI: 1.001–1.006, *p* = 0.006; SII 45: OR: 1.002, 95% CI: 1.001–1.003, *p* < 0.001; SIRI 45: OR: 1.283, 95% CI: 1.103–1.492, *p* = 0.001). At the 90th minute during cardiopulmonary bypass measurement, SII and SIRI were significantly associated with mortality (SII 90: OR: 1.001, 95% CI: 1.000–1.001, *p* = 0.009; SIRI 90: OR: 1.163, 95% CI: 1.020–1.327, *p* = 0.025). Postoperative NLR, PLR, SII, and SIRI were significantly associated with mortality, with odds ratios below 1 (NLR post: OR: 0.851, 95% CI: 0.794–0.913, *p* < 0.001; PLR post: OR: 0.989, 95% CI: 0.985–0.993, *p* < 0.001; SII post: OR: 0.999, 95% CI: 0.999–1.000, *p* < 0.001; SIRI post: OR: 0.924, 95% CI: 0.877–0.973, *p* = 0.003) (Table 6).

The multivariable logistic regression analysis results are presented in Table 7. Age was independently associated with mortality across all models. Among the inflammatory indices, NLR measured at the 5th minute was independently associated with mortality in Model B and Model E, whereas SII measured at the 5th minute remained independently associated with mortality in Model C, Model E, and Model G. Similarly, SIRI measured at the 5th minute was independently associated with mortality in Model D and Model F. The discrimination performance of the models improved after the addition of inflammatory indices to the limited study-specific clinical model. The clinical model, which included age, ejection fraction, and preoperative creatinine, demonstrated an AUC of 0.698, whereas the addition of 5th-minute SII increased the AUC to 0.797. A formal comparison of correlated ROC curves using DeLong’s test confirmed a significant improvement in discrimination for the limited clinical model plus 5th-minute SII compared with the limited clinical model alone. However, because established operative risk scores such as STS and EuroSCORE II were not incorporated, this improvement should be interpreted as incremental discrimination beyond the limited study-specific clinical model, not beyond contemporary standard surgical risk assessment (ΔAUC = 0.099; DeLong *p* = 0.0007). Similarly, the addition of 5th-minute SIRI improved model discrimination compared with the clinical model alone (AUC increase from 0.698 to 0.787; ΔAUC = 0.089; DeLong *p* = 0.0055). The highest apparent AUC was observed in the secondary exploratory Model E, which included the clinical variables, NLR 5 min, and SII 5 min (AUC = 0.821). Bootstrap internal validation demonstrated limited optimism, with the optimism-corrected AUC of Model E remaining above 0.80. Calibration assessment demonstrated acceptable apparent model fit, with calibration intercepts close to zero, calibration slopes close to one, and Hosmer–Lemeshow *p* values > 0.05 across the models. Multicollinearity analysis demonstrated acceptable variance inflation factor values in the primary models, whereas the secondary exploratory combination models including multiple inflammatory indices showed higher VIF values, consistent with shared biological components. The reversal of the NLR coefficient direction in the model combining NLR and SII was therefore interpreted cautiously and was considered more likely to reflect shared variance and multicollinearity between related inflammatory indices rather than a true inverse biological association. A post hoc power analysis based on the primary SII 5 min model demonstrated an achieved statistical power exceeding 95%. However, because of the enriched nested case-control design and the absence of external validation, these discrimination, calibration, and incremental performance findings should be interpreted as exploratory rather than as externally validated estimates of clinical prediction performance (Table 7).

In the sensitivity analyses that additionally included cardiopulmonary bypass duration and aortic cross-clamp time, the overall discriminative performance of the models increased, as expected. However, the direction of the associations between the inflammatory indices and mortality remained generally consistent. These findings suggest that the prognostic information provided by the inflammatory indices is not solely explained by the operative duration variables (Table 8).

The ROC analysis results for mortality prediction are presented in Table 8. At the baseline measurement, SII demonstrated the highest discriminative performance (AUC: 0.698), followed by PLR (AUC: 0.649), NLR (AUC: 0.592), and SIRI (AUC: 0.586). At the 5 min CPB measurement, SII showed the highest predictive performance (AUC: 0.790), followed by SIRI (AUC: 0.765), PLR (AUC: 0.687), and NLR (AUC: 0.681). At the 45th minute during cardiopulmonary bypass measurement, SII demonstrated the highest discriminative ability (AUC: 0.737), followed by SIRI (AUC: 0.693), PLR (AUC: 0.649), and NLR (AUC: 0.563). At the 90th minute during cardiopulmonary bypass measurement, PLR showed the highest AUC value (AUC: 0.589), followed by NLR (AUC: 0.574), SIRI (AUC: 0.542), and SII (AUC: 0.515). In the postoperative measurement, PLR demonstrated the highest predictive performance (AUC: 0.789), followed by NLR (AUC: 0.757), SII (AUC: 0.711), and SIRI (AUC: 0.628) (Table 9).

**Figure 2:**
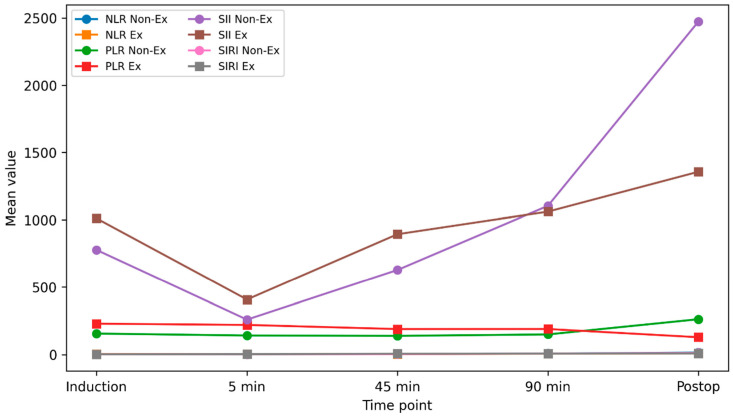
Dynamic changes in inflammatory indices across during cardiopulmonary bypass and postoperative time points according to mortality status. Figure 1 illustrates the temporal changes in inflammatory indices, including neutrophil-to-lymphocyte ratio (NLR), platelet-to-lymphocyte ratio (PLR), systemic immune-inflammation index (SII), and Systemic Inflammatory Response Index (SIRI), measured at induction (baseline), during cardiopulmonary bypass 5th, 45th, and 90th minutes, and postoperative period, stratified by mortality status. Mean values are presented for both mortality (Ex) and survival (Non-Ex) groups. The figure demonstrates distinct temporal patterns between groups, with differences in during cardiopulmonary bypass and postoperative trajectories. Ex = Mortality occurred, Non-Ex = Mortality did not occur, NLR = Neutrophil-to-Lymphocyte Ratio, PLR = Platelet-to-Lymphocyte Ratio, SII = Systemic Immune-Inflammation Index, SIRI = Systemic Inflammatory Response Index.

The temporal distribution of inflammatory indices according to mortality status is presented in Figure 2. At baseline, SII values were higher in patients who experienced mortality compared with survivors, and this difference persisted across all during-cardiopulmonary-bypass time points. Similarly, PLR values were consistently higher in the mortality group during cardiopulmonary bypass measurements. SIRI values were also elevated in the mortality group at all measured time points. In contrast, the postoperative measurements showed higher NLR, PLR, SII, and SIRI values in the survival group compared to the mortality group. All indices demonstrated dynamic changes over time, with distinct temporal patterns observed between the mortality and survival groups (Figure 2).

**Figure 3:**
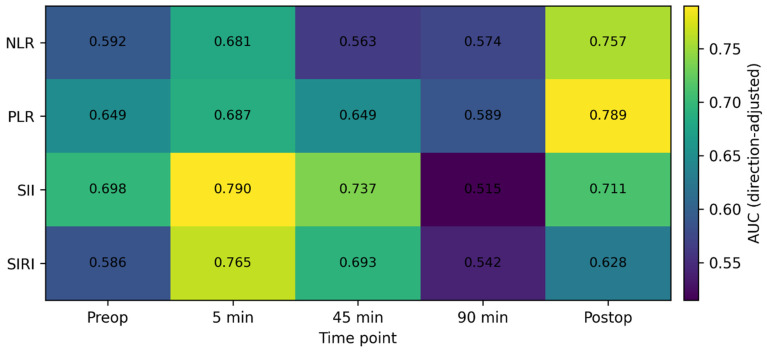
Heatmap of AUC values for inflammatory indices across perioperative time points. Figure 2 presents a heatmap illustrating the area under the curve (AUC) values derived from receiver operating characteristic (ROC) analysis for inflammatory indices, including neutrophil-to-lymphocyte ratio (NLR), platelet-to-lymphocyte ratio (PLR), systemic immune-inflammation index (SII), and Systemic Inflammatory Response Index (SIRI), across different perioperative time points (preoperative baseline, during cardiopulmonary bypass 5th, 45th, and 90th minutes, and postoperative period). Each cell represents the AUC value corresponding to a specific marker and time-point, with color intensity reflecting the magnitude of discriminative performance. Higher AUC values indicate better predictive accuracy for in-hospital mortality. AUC = Area Under the Curve, ROC = Receiver Operating Characteristic, NLR = Neutrophil-to-Lymphocyte Ratio, PLR = Platelet-to-Lymphocyte Ratio, SII = Systemic Immune-Inflammation Index, SIRI = Systemic Inflammatory Response Index, Preop = Preoperative baseline measurement, Postop = Postoperative measurement.

The heatmap illustrating AUC values of inflammatory indices across perioperative time points is presented in Figure 3. At baseline, SII demonstrated the highest predictive performance (AUC: 0.698), followed by PLR (AUC: 0.649), NLR (AUC: 0.592), and SIRI (AUC: 0.586). At the 5 min CPB measurement, SII showed the highest AUC value (AUC: 0.790), followed by SIRI (AUC: 0.765), PLR (AUC: 0.687), and NLR (AUC: 0.681). At the 45th minute during cardiopulmonary time point bypass, SII demonstrated the highest predictive performance (AUC: 0.737), followed by SIRI (AUC: 0.693), PLR (AUC: 0.649), and NLR (AUC: 0.563). At the 90th minute during cardiopulmonary bypass measurement, PLR had the highest AUC value (AUC: 0.589), followed by NLR (AUC: 0.574), SIRI (AUC: 0.542), and SII (AUC: 0.515). In the postoperative period, PLR demonstrated the highest predictive performance (AUC: 0.789), followed by NLR (AUC: 0.757), SII (AUC: 0.711), and SIRI (AUC: 0.628) (Figure 3).

**Figure 4:**
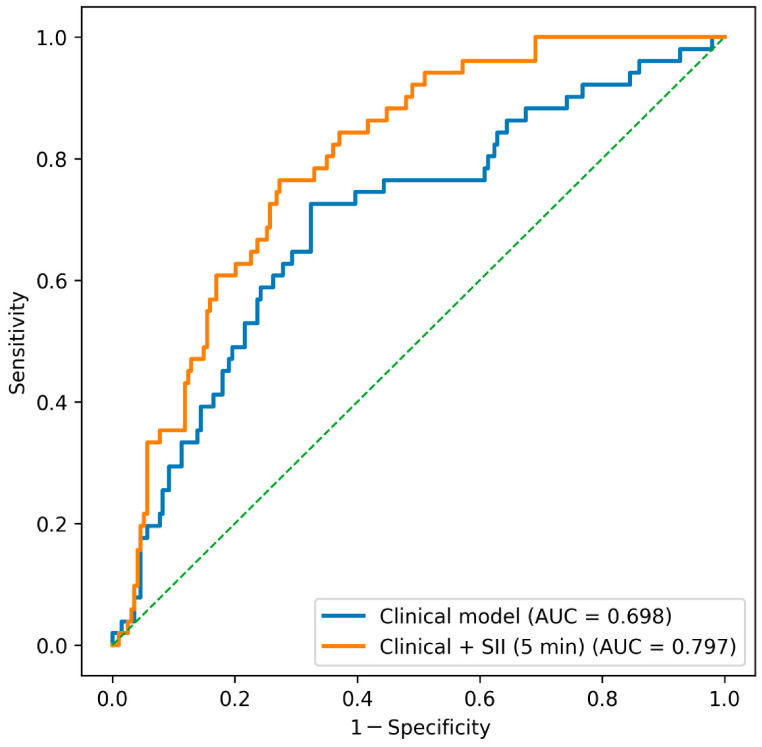
Exploratory incremental predictive performance of SII measured at the 5th minute beyond the limited study-specific clinical model for in-hospital mortality. Receiver operating characteristic curves comparing the clinical model alone and the clinical model combined with systemic immune-inflammation index (SII) measured at the 5th minute during cardiopulmonary bypass. The clinical model included age, ejection fraction, and preoperative creatinine. The addition of 5th-minute SII increased the AUC from 0.698 to 0.797. Formal comparison of correlated ROC curves using DeLong’s test demonstrated a significant improvement in discrimination (ΔAUC = 0.099; DeLong *p* = 0.0007). These findings should be interpreted as exploratory and as reflecting incremental discrimination beyond the limited study-specific clinical model, because established operative risk scores such as STS and EuroSCORE II were not incorporated. Therefore, these results should not be interpreted as incremental value beyond contemporary standard surgical risk assessment. ROC = receiver operating characteristic; AUC = area under the curve; SII = systemic immune-inflammation index.

The ROC curve analysis comparing the clinical model and the clinical model combined with SII measured at the 5th minute is presented in Figure 4. The clinical model demonstrated an AUC of 0.698 for predicting in-hospital mortality. The addition of SII measured at the 5th minute increased the AUC from 0.698 to 0.797. A formal comparison of correlated ROC curves using DeLong’s test confirmed a significant improvement in discrimination (ΔAUC = 0.099; DeLong *p* = 0.0007). Calibration assessment demonstrated acceptable apparent model fit, although these findings should be interpreted as exploratory because of the enriched nested case–control design and the absence of external validation (Table 6 and Figure 4).

## 4. Discussion

In this study, we demonstrated that the dynamic evaluation of inflammatory indices during cardiopulmonary bypass is strongly and time-dependently associated with in-hospital mortality. The most notable finding was that the systemic immune-inflammation index and the systemic inflammatory response index showed higher and more consistent prognostic performance in predicting mortality compared with the traditionally used neutrophil-to-lymphocyte ratio and platelet-to-lymphocyte ratio. This finding indicates that the inflammatory process occurring during cardiopulmonary bypass may be more accurately represented not only by the neutrophil–lymphocyte balance but also by integrating platelet- and monocyte-mediated responses.

The prognostic value of NLR and PLR in cardiac surgery and cardiovascular diseases has been well established [3]. In particular, meta-analyses have demonstrated that perioperative NLR is independently associated with mortality [12]. However, most of these studies evaluated the indices at a single preoperative or postoperative time point. In contrast, our study analyzed these indices dynamically during cardiopulmonary bypass and demonstrated that the early during-cardiopulmonary-bypass phase is particularly critical from a prognostic perspective. In this regard, our study differs from the static measurement approach commonly used in the current literature.

In recent years, SII has been shown to be a stronger prognostic marker than NLR and PLR in cardiovascular diseases [13]. Karadeniz et al. reported that SII was independently associated with mortality in patients with acute coronary syndrome and demonstrated higher discriminative performance compared with NLR [14]. Similarly, Wang et al. showed that SII and SIRI may serve as superior risk markers compared with NLR and PLR, as they incorporate both inflammatory and thrombotic components [15]. Consistent with these findings, the association between SII and mortality observed in our study, as well as the improvement in exploratory discrimination when added to the limited study-specific clinical model, supports the concept that SII may reflect the complex inflammatory response occurring during cardiopulmonary bypass. However, because established operative risk scores such as STS and EuroSCORE II were not available, this finding should not be interpreted as demonstrating incremental value beyond contemporary standard surgical risk assessment. The unique contribution of our study lies in evaluating this association during the early cardiopulmonary bypass period.

Although the literature on SIRI remains more limited, indices incorporating the monocyte component have been reported to show a strong association with mortality and organ dysfunction [16]. Since monocytes play a key role in the sustained phase of the systemic inflammatory response, SIRI has been proposed as a more advanced indicator of inflammatory burden [15]. Studies conducted in intensive care and cardiac surgery populations have reported that SIRI may serve as a stronger predictor of mortality compared with NLR [8]. In our study, the significant association between SIRI and mortality is consistent with these findings; however, the more stable and higher prognostic performance of SII compared with SIRI suggests that the platelet component may play a central role in the thromboinflammatory process occurring during cardiopulmonary bypass. In this respect, our results provide a more comprehensive framework by incorporating the platelet dimension into the predominantly monocyte-focused perspective in the literature.

Comparative analyses of inflammatory indices remain limited in the existing literature. Most studies have evaluated a single index. One of the major contributions of our study is the comparative analysis of NLR, PLR, SII, and SIRI within the same cohort, at the same point, and within the same statistical model. This direct comparison suggested stronger exploratory discrimination of SII and SIRI compared with traditional indices within the present enriched analytical sample. In particular, the increase in discriminative performance following the addition of inflammatory indices to the limited study-specific clinical model suggests that these parameters may provide complementary prognostic information within this dataset. However, because STS and EuroSCORE II were not incorporated, the present findings should not be interpreted as evidence that these indices add incremental value beyond established surgical risk scores.

Another important finding is that the inflammatory indices showed a strong association with mortality, particularly during the early phase of cardiopulmonary bypass. The superior prognostic performance observed at the 5th minute of cardiopulmonary bypass may be biologically plausible. This early time point likely captures the immediate host response following blood contact with the artificial surfaces of the extracorporeal circuit, which rapidly triggers complement activation, neutrophil recruitment, platelet activation, and cytokine release. Patients who subsequently experienced mortality may have exhibited a disproportionately amplified inflammatory response during this initial phase, resulting in an early divergence of the inflammatory indices from those of survivors. In contrast, measurements obtained later during cardiopulmonary bypass may be increasingly influenced by hemodilution, temperature management, transfusion exposure, ultrafiltration practices, pharmacological interventions, and progressive physiological adaptation to extracorporeal circulation. These factors may attenuate the discriminative capacity of inflammatory markers over time. Therefore, the 5th-minute measurements may represent a particularly sensitive window for identifying patients with an exaggerated biological response to cardiopulmonary bypass and a consequently higher risk of adverse outcomes. It should also be considered that the inflammatory indices measured at the 5th minute may partially reflect the pre-existing inflammatory status rather than solely CPB-induced changes. However, the rapid divergence observed between mortality and survival groups suggests that early during-cardiopulmonary-bypass measurements capture both baseline inflammatory burden and the initial response to cardiopulmonary bypass. The baseline-adjusted change analyses further support a cautious interpretation of the dynamic inflammatory response during cardiopulmonary bypass. Not all raw between-group differences persisted when evaluated as absolute or percentage changes from the induction values, suggesting that early CPB measurements may be influenced by the baseline inflammatory status, hemodilution, circuit priming, and hematologic redistribution. Nevertheless, the greater baseline-adjusted increase in SIRI and the less pronounced percentage reduction in SII at the 5th minute among the mortality cases suggest that at least part of the early divergence may reflect a differential host inflammatory response during the initial phase of cardiopulmonary bypass. Importantly, these findings should not be interpreted as evidence that the inflammatory indices act independently from operative complexity or cardiopulmonary bypass burden alone. In the present study, the exploratory sensitivity analyses incorporating cardiopulmonary bypass duration and aortic cross-clamp time demonstrated generally consistent directions of association between inflammatory indices and mortality. However, these findings do not exclude residual confounding related to operative duration, technical complexity, or perioperative physiological instability. Cardiopulmonary bypass duration and aortic cross-clamp time are not only measures of operative exposure but may also act as surrogate markers of procedural difficulty, diffuse coronary disease, incomplete hemodynamic reserve, intraoperative instability, myocardial ischemia–reperfusion burden, and postoperative organ dysfunction risk. Therefore, the observed associations between the inflammatory indices and mortality should not be interpreted as fully independent of the operative burden. Instead, these biomarkers should be considered as nonspecific integrative markers that may reflect both the host inflammatory response and the cumulative physiological stress imposed by surgery and extracorporeal circulation. Consequently, the observed prognostic value of the inflammatory indices should be interpreted within the broader context of the operative burden and perioperative risk. Therefore, the inflammatory indices likely reflect the integrated biological response generated by these perioperative stressors rather than isolated inflammatory pathways alone. Nevertheless, the persistence of significant associations after adjustment for the variables included in the study-specific models suggests that the magnitude of the inflammatory response may provide exploratory prognostic information within the present analytical framework. Previous studies have generally evaluated the consequences of the inflammatory response, whereas our study demonstrated that the inflammatory response provides prognostic signals during the cardiopulmonary bypass period when it is still developing. This finding suggests that early identification of inflammatory activation may potentially influence clinical decision-making processes. A particularly important aspect of our findings is the paradoxically lower postoperative NLR, PLR, SII, and SIRI values in patients who experienced mortality compared with survivors, despite markedly higher during-cardiopulmonary-bypass levels. Several mechanisms may explain this apparently paradoxical finding. First, survivor bias should be considered, as patients who died early after surgery may not have survived long enough to develop the delayed postoperative inflammatory rebound often observed in critically ill but recovering patients. Second, postoperative hemodilution related to fluid administration, bleeding, transfusion requirements, and renal replacement therapies may have altered circulating leukocyte and platelet concentrations. Third, an exaggerated early inflammatory response may be followed by immune exhaustion or immunologic paralysis, characterized by impaired leukocyte responsiveness and relative suppression of the inflammatory indices despite ongoing clinical deterioration. In addition, an accelerated consumption, sequestration, or redistribution of neutrophils, lymphocytes, monocytes, and platelets during severe systemic inflammation may contribute to lower measured postoperative values. Finally, the differences in transfusion burden and postoperative critical care interventions may further influence hematological parameters and inflammatory index calculations. Therefore, lower postoperative inflammatory indices should not necessarily be interpreted as evidence of a less severe inflammatory state, but rather as a potential marker of dysregulated or exhausted immune response in patients with poor outcomes. This reversal pattern warrants a more nuanced interpretation than a simple immune paralysis hypothesis alone. First, survivor bias is a major consideration, as patients who died early after surgery may not have survived long enough to develop the delayed postoperative inflammatory rebound typically observed in critically ill but recovering patients. Second, although the postoperative blood sampling time was standardized to the 24th postoperative hour, variability in the exact clinical context of sampling—including proximity to hemodynamic deterioration, organ support escalation, transfusion exposure, or emergent reintervention—may still have influenced the measured indices. Third, differences in postoperative intensive care management, including vasopressor requirements, transfusion burden, renal replacement therapy, or infection prophylaxis, may have modified leukocyte and platelet kinetics differently between groups. In addition, several postoperative laboratory abnormalities observed in the mortality group, including sodium imbalance, elevated liver enzymes, anemia, and renal dysfunction, are expected findings in critically ill patients following major cardiac surgery and likely reflect the severity of postoperative physiological deterioration rather than specific inflammatory mechanisms alone. Therefore, these postoperative biochemical findings should primarily be interpreted as supportive markers of overall postoperative clinical severity. Consequently, these associations should not be interpreted as evidence of independent prognostic utility or causal involvement in mortality. Rather, they are more likely to reflect downstream manifestations of postoperative organ dysfunction and physiological deterioration occurring after surgery. This inverse postoperative direction was also considered in the ROC analyses. Therefore, the postoperative AUC values should not be interpreted as indicating that higher postoperative inflammatory indices predicted mortality. Rather, they reflect the ability of lower postoperative NLR, PLR, SII, and SIRI values to discriminate mortality cases, consistent with the odds ratios below 1 observed in the logistic regression analyses.

In addition, the lower postoperative indices observed in the mortality group should be interpreted cautiously and should not be regarded as specific evidence of immune exhaustion. Although immune exhaustion following an early excessive cardiopulmonary bypass-related inflammatory response remains a possible explanation, the postoperative inflammatory indices may also be substantially influenced by sampling conditions, transfusion exposure, hemodilution, fluid administration, renal replacement therapy, perioperative physiological deterioration, and differences in intensive care management. Therefore, the postoperative NLR, PLR, SII, and SIRI values should be considered nonspecific markers reflecting a complex interaction between inflammatory activity, hematologic redistribution, treatment-related effects, and overall clinical severity. This trajectory is biologically plausible in cardiopulmonary bypass-related systemic inflammation and may indicate a failure to sustain an adaptive inflammatory recovery phase. Nevertheless, this finding should also be interpreted as an important study limitation, as the residual bias related to postoperative timing, the survivor effect, and differences in postoperative care cannot be fully excluded. Furthermore, although the internal validation was performed using bootstrap resampling techniques, an external validation in independent patient populations was not available. Therefore, the generalizability of the observed prognostic performance remains uncertain, and the findings should be confirmed in multicenter prospective cohorts before a broader clinical application is considered. In addition, because multiple inflammatory indices were evaluated across several perioperative time points without a formal correction for multiple testing, the findings should be considered exploratory and hypothesis-generating. Accordingly, individual statistically significant associations should be interpreted cautiously until confirmed in independent prospective cohorts. The detection of excessive inflammatory activation during the cardiopulmonary bypass period may have potential value for early intraoperative risk evaluation and for planning closer postoperative monitoring. However, the present study did not evaluate the laboratory turnaround time, automated calculation workflows, intraoperative decision algorithms, or whether these indices can be acted upon during surgery. Therefore, any near-real-time clinical application of these indices remains hypothetical and requires prospective validation before integration into perioperative decision-making pathways.

In conclusion, our study suggests that the dynamic and comparative evaluation of the inflammatory indices during cardiopulmonary bypass may provide exploratory prognostic information regarding in-hospital mortality. In particular, SII and SIRI showed stronger discrimination than the traditional indices within the present enriched analytical sample. The addition of the 5th-minute SII improved discrimination beyond the limited study-specific clinical model including age, ejection fraction, and preoperative creatinine. However, because established operative risk scores such as STS and EuroSCORE II were not incorporated, this improvement should not be interpreted as incremental value beyond contemporary standard surgical risk assessment. Given the retrospective single-center design, enriched nested case–control sampling, and absence of external validation, these findings should be considered exploratory and hypothesis-generating. Prospective multicenter studies are required to confirm the reproducibility, generalizability, and potential clinical applicability of these observations.

### Limitations of the Study

This study has several important limitations that should be acknowledged. First, due to its retrospective and observational design, causal relationships between the inflammatory indices and in-hospital mortality cannot be definitively established. The associations observed between SII, SIRI, NLR, PLR, and mortality reflect prognostic correlations rather than direct causal effects. Prospective studies are required to determine whether these biomarkers can be used to guide clinical interventions and improve outcomes. Another important limitation is that the clinical base model included only age, ejection fraction, and preoperative creatinine. Established operative risk scores such as STS and EuroSCORE II were not consistently available in the retrospective dataset and therefore could not be incorporated into the primary adjustment or incremental discrimination analyses. Consequently, the observed improvement in discrimination after adding the 5th-minute SII should be interpreted as an incremental value beyond this limited study-specific clinical model, rather than beyond contemporary standard surgical risk assessment. Future studies should evaluate whether intraoperative inflammatory indices provide additional prognostic value when integrated with validated surgical risk scores.

Second, the study cohort was intentionally constructed using a nested case–control framework with an overrepresentation of mortality cases to enhance statistical power and allow balanced comparative analysis. Therefore, the observed mortality proportion does not reflect the true institutional mortality rate. This design improves analytical robustness but limits the ability to extrapolate absolute risk estimates to the general cardiac surgery population.

Third, this was a single-center study conducted at a tertiary academic institution. Although this ensured consistency in surgical techniques, cardiopulmonary bypass protocols, and laboratory measurements, it may limit the generalizability of the findings to other centers with different patient populations, surgical practices, and perioperative management strategies. Multicenter studies are necessary to validate the external applicability of these findings.

Fourth, although multiple inflammatory indices were analyzed and directly compared, additional inflammatory and immunologic biomarkers such as interleukin-6, tumor necrosis factor-alpha, procalcitonin, and complement activation markers were not available for analysis. The inclusion of these biomarkers could have provided a more comprehensive characterization of the inflammatory response and further clarified the mechanistic relationships between systemic inflammation and mortality.

Fifth, although the serial measurements provided important insight into the dynamic inflammatory response during cardiopulmonary bypass, a postoperative longitudinal monitoring beyond the immediate perioperative period was not performed. Moreover, the paradoxical reversal of the postoperative inflammatory indices should be interpreted cautiously, as the survivor bias, postoperative care heterogeneity, and timing-related residual confounding may have influenced these late measurements. Therefore, the long-term prognostic value of these indices could not be assessed.

Sixth, because the serial inflammatory indices were obtained repeatedly from the same patients, the use of separate time point-specific regression models did not fully account for within-subject dependency. Although this analytical strategy was selected to evaluate clinically interpretable perioperative decision windows, the residual correlation between repeated measurements may have affected precision estimates and should be considered when interpreting temporal comparisons. Future prospective studies should confirm these findings using longitudinal mixed-effects models or generalized estimating equation approaches.

Finally, although this study demonstrated the superior prognostic performance of SII and SIRI compared with traditional indices such as NLR and PLR, optimal threshold values for clinical decision-making were not definitively established. Future large-scale, prospective, and multicenter studies are needed to validate these findings, define clinically actionable cut-off values, and determine whether real-time integration of these indices into perioperative risk stratification models can improve patient outcomes.

## 5. Conclusions

This study demonstrates that the inflammatory response triggered during cardiopulmonary bypass is not merely a physiological bystander phenomenon but a measurable and prognostically powerful process. Among the evaluated indices, SII and SIRI consistently outperformed traditional markers such as NLR and PLR in predicting in-hospital mortality. By integrating neutrophil activation, lymphocyte suppression, platelet-driven thromboinflammation, and monocyte-mediated cytokine response into composite indices, SII and SIRI more accurately captured the multidimensional inflammatory burden of cardiopulmonary bypass. Importantly, their prognostic signal emerged during the early cardiopulmonary bypass phase, suggesting that the mortality risk becomes biologically detectable before clinical deterioration manifests.

These findings suggest that the dynamic intraoperative assessment of comprehensive inflammatory indices may provide exploratory prognostic information during cardiopulmonary bypass. However, the observed improvement in discrimination after adding 5th-minute SII should be interpreted as an incremental value beyond a limited study-specific clinical model, rather than beyond established operative risk scores such as STS or EuroSCORE II. Therefore, these indices should not be considered alternatives to contemporary surgical risk assessment. Future prospective and multicenter studies are warranted to determine whether integrating SII and SIRI with validated operative risk models can improve perioperative risk stratification or clinical decision-making. The relationship between these inflammatory indices and mortality was clearly time-dependent, and the complex postoperative trajectory observed in this study warrants further mechanistic and prospective investigation. Given the nested case–control design and intentional event oversampling, these findings should not be interpreted as definitive clinical prediction tools but rather as exploratory evidence supporting future validation studies. Future prospective and multicenter studies are warranted to validate threshold values and determine whether integrating these indices into structured perioperative algorithms can translate into measurable improvements in surgical outcomes. The relationship between these inflammatory indices and mortality was clearly time-dependent, and the complex postoperative trajectory observed in this study warrants further mechanistic and prospective investigation. Given the nested case–control design and intentional event oversampling, these findings should not be interpreted as definitive clinical prediction tools but rather as exploratory evidence supporting future validation studies.

### 5.1. Key Points

#### 5.1.1. What Is Known About the Topic?

Systemic inflammation is a fundamental pathophysiological component of cardiopulmonary bypass (CPB) and plays a central role in determining postoperative outcomes after coronary artery bypass grafting (CABG). Traditional hematological inflammatory indices such as the neutrophil-to-lymphocyte ratio (NLR) and the platelet-to-lymphocyte ratio (PLR) have been widely investigated and shown to be associated with adverse outcomes and mortality in cardiovascular surgery and other cardiovascular diseases. However, most previous studies evaluated these indices at a single preoperative or postoperative time points, limiting their ability to reflect the dynamic and multidimensional inflammatory response that develops during CPB. More recently, composite inflammatory indices such as the systemic immune-inflammation index (SII) and the systemic inflammatory response index (SIRI), which integrate neutrophil, platelet, monocyte, and lymphocyte responses, have emerged as more comprehensive markers of the systemic inflammatory burden.

#### 5.1.2. What Does This Study Add?

This study demonstrates that SII and SIRI provide more consistent and clinically relevant prognostic performance than traditional inflammatory indices such as NLR and PLR for predicting in-hospital mortality in patients undergoing elective isolated CABG with CPB. By comparatively evaluating multiple inflammatory indices across serial intraoperative CPB time points, the study shows that composite indices integrating multiple inflammatory cell lineages may better reflect the biological magnitude of perioperative inflammatory activation. Importantly, the prognostic signal of these indices became apparent during the early intraoperative CPB period, suggesting their potential utility as near-real-time indicators of increased mortality risk. These findings support the concept that the dynamic monitoring of the inflammatory burden during CPB may provide exploratory prognostic information in elective isolated CABG surgery; however, its incremental value beyond established operative risk scores requires prospective validation.

## Figures and Tables

**Table 1:** Baseline characteristics and operative data of the enriched analytical sample (51 mortality cases and 194 sampled surviving controls) (*n* = 245).

Variable	Overall (Mean ± SD/*n*, %)	Median (Min–Max)
Age (years)	59.3 ± 12.5	61 (19–80)
Male, *n* (%)	156 (63.7)	
Female, *n* (%)	89 (36.3)	
Mortality (Ex), *n* (%)	51 (20.8)	
Survived (Non-Ex), *n* (%)	194 (79.2)	
DM yes, *n* (%)	125 (51.0)	
HT yes, *n* (%)	147 (60.0)	
Pump time (min)	117.2 ± 44.3	110 (36–254)
Cross-clamp time (min)	69.1 ± 29.1	62 (12–143)

Statistical tests applied include assessment of normality using the Kolmogorov–Smirnov test. Continuous variables with normal distribution are presented as mean ± standard deviation (SD) and were analyzed using the independent samples Student’s *t*-test, while continuous variables without normal distribution are presented as median (minimum–maximum) and were analyzed using the Mann–Whitney U test. Categorical variables are expressed as number (*n*) and percentage (%) and were analyzed using the Chi-square test or Fisher’s exact test, as appropriate. Because the study used an enriched nested case–control design, the mortality proportion shown in this table reflects the composition of the analytical sample rather than the institutional mortality rate. In the eligible elective isolated on-pump CABG source population, 51 of 874 patients experienced in-hospital mortality, whereas the final analytical sample intentionally included all mortality cases and a randomly sampled subset of surviving controls. Therefore, the mortality proportion in Table 1 should not be extrapolated to the overall eligible CABG source population. Ex = Mortality occurred, Non-Ex = Mortality did not occur, DM = Diabetes Mellitus, HT = Hypertension, SD = Standard Deviation, min = Minimum, max = Maximum.

**Table 2:** Preoperative and postoperative laboratory parameters by mortality status.

Parameter	Non-Ex (Mean ± SD)	Ex (Mean ± SD)	*p*
EF	47.80 ± 10.86	46.90 ± 10.86	0.599
Pre-Na	138.97 ± 2.25	138.80 ± 1.79	0.581
Post-Na	138.07 ± 2.80	136.63 ± 2.64	**<0.001**
Pre-K	4.23 ± 0.36	4.16 ± 0.36	0.217
Post-K	4.38 ± 0.45	4.44 ± 0.68	0.561
Pre-ALT	19.22 ± 9.56	18.75 ± 8.79	0.741
Post-ALT	24.86 ± 12.71	29.33 ± 15.41	**0.038**
Pre-AST	24.41 ± 10.63	23.75 ± 10.24	0.695
Post-AST	35.96 ± 16.34	47.57 ± 20.53	**<0.001**
Pre-CRP	8.55 ± 7.11	9.32 ± 7.62	0.517
Post-CRP	66.86 ± 23.64	84.28 ± 26.69	**<0.001**
Pre-HB	12.64 ± 1.63	12.42 ± 1.72	0.392
Post-HB	10.52 ± 1.45	9.77 ± 1.61	**0.002**
Pre-WBC	8.29 ± 2.22	8.53 ± 2.18	0.468
Post-WBC	12.41 ± 3.27	14.92 ± 3.91	**<0.001**
Pre-Creatinine	1.22 ± 0.51	1.34 ± 0.61	0.171
Post-Creatinine	1.39 ± 0.70	1.98 ± 1.07	**<0.001**
neut-ind	6.08 ± 1.86	6.53 ± 1.84	0.126
lymph-ind	2.06 ± 0.64	1.76 ± 0.57	**0.002**
plt-ind	233.71 ± 55.68	236.49 ± 63.87	0.759
Monosit-ind	0.53 ± 0.18	0.55 ± 0.17	0.471
neut-postop	10.10 ± 2.97	12.40 ± 3.44	**<0.001**
lymph-postop	1.42 ± 0.51	1.08 ± 0.40	**<0.001**
plt-post	206.59 ± 50.97	171.51 ± 47.92	**<0.001**
Monosit-postop	0.67 ± 0.23	0.86 ± 0.28	**<0.001**

Statistical tests applied include assessment of normality using the Kolmogorov–Smirnov test. Continuous variables are presented as mean ± standard deviation (SD) and were analyzed using the independent samples Student’s *t*-test for normally distributed data and the Mann–Whitney U test for non-normally distributed data, as appropriate. Categorical variables, when applicable, were analyzed using the Chi-square test or Fisher’s exact test. Preoperative and postoperative laboratory parameters were compared between mortality and survival groups using independent samples analysis. In the table, statistically significant values are marked in bold. The *p*-value indicates the level of statistical significance, where values less than 0.05 are considered statistically significant. Ex = Mortality occurred, Non-Ex = Mortality did not occur, EF = Ejection Fraction, Na = Sodium, K = Potassium, ALT = Alanine Aminotransferase, AST = Aspartate Aminotransferase, CRP = C-Reactive Protein, HB = Hemoglobin, WBC = White Blood Cell Count, neut-ind = Preoperative Neutrophils, lymph-ind = Preoperative Lymphocytes, plt-ind = Preoperative Platelets, Monosit-ind = Preoperative Monocytes, neut-postop = Postoperative Neutrophils, lymph-postop = Postoperative Lymphocytes, plt-post = Postoperative Platelets, Monosit-postop = Postoperative Monocytes, SD = Standard Deviation.

**Table 3:** Dynamic during cardiopulmonary bypass indices by time point and mortality.

Index/Time Point	Non-Ex (Mean ± SD)	Ex (Mean ± SD)	*p*
NLR ind	3.37 ± 2.06	3.96 ± 1.97	0.060
PLR ind	154.70 ± 76.82	228.26 ± 132.23	**<0.001**
SII ind	776.56 ± 594.64	1010.50 ± 411.62	**0.001**
SIRI ind	2.03 ± 1.43	2.41 ± 1.53	0.114
NLR 5	2.17 ± 1.19	2.84 ± 0.99	**<0.001**
PLR 5	140.49 ± 64.76	218.94 ± 116.78	**<0.001**
SII 5	258.17 ± 180.62	407.00 ± 132.64	**<0.001**
SIRI 5	2.03 ± 1.33	3.40 ± 1.64	**<0.001**
NLR 45	2.45 ± 1.25	2.63 ± 1.14	0.291
PLR 45	166.32 ± 69.09	217.47 ± 101.23	**<0.001**
SII 45	388.64 ± 212.34	585.50 ± 278.05	**<0.001**
SIRI 45	2.06 ± 1.06	2.86 ± 1.27	**<0.001**
NLR 90	2.77 ± 1.38	3.15 ± 1.48	0.085
PLR 90	189.60 ± 74.54	220.77 ± 93.59	**0.020**
SII 90	489.05 ± 272.72	655.78 ± 330.90	**<0.001**
SIRI 90	2.15 ± 1.08	2.72 ± 1.18	**0.003**
NLR post	14.87 ± 9.09	8.27 ± 4.86	**<0.001**
PLR post	261.33 ± 161.58	127.64 ± 92.97	**<0.001**
SII post	2473.81 ± 1676.08	1356.57 ± 731.41	**<0.001**
SIRI post	12.70 ± 8.64	8.63 ± 5.61	**<0.001**

Statistical tests applied include assessment of normality using the Kolmogorov–Smirnov test. Continuous variables are presented as mean ± standard deviation (SD) and were analyzed using the independent samples Student’s *t*-test for normally distributed data and the Mann–Whitney U test for non-normally distributed data, as appropriate. dynamically changing inflammatory indices during cardiopulmonary bypass measured at different time points were compared between mortality and survival groups using independent samples analysis. In the table, statistically significant values are marked in bold. The *p*-value indicates the level of statistical significance, where values less than 0.05 are considered statistically significant. Analyses for the 90 min time-point were restricted to patients who remained on cardiopulmonary bypass for ≥90 min (*n* = 184). Ex = Mortality occurred, Non-Ex = Mortality did not occur, NLR = Neutrophil-to-Lymphocyte Ratio, PLR = Platelet-to-Lymphocyte Ratio, SII = Systemic Immune-Inflammation Index, SIRI = Systemic Inflammatory Response Index, ind = Baseline (Induction), 5 = 5th minute during cardiopulmonary bypass measurement, 45 = 45th minute during cardiopulmonary bypass measurement, 90 = 90th minute during cardiopulmonary bypass measurement, post = Postoperative measurement, SD = Standard Deviation.

**Table 4:** Baseline-adjusted absolute and percentage changes in inflammatory indices during cardiopulmonary bypass according to mortality status.

Index	Time Point	Absolute Change Non-Ex	Absolute Change Ex	*p*	Percentage Change Non-Ex	Percentage Change Ex	*p*
NLR	5 min	−1.24 ± 1.60	−1.13 ± 1.94	0.692	−28.7 ± 41.0	−17.0 ± 41.2	0.074
NLR	45 min	−0.12 ± 1.77	0.68 ± 3.42	0.109	16.6 ± 58.8	39.2 ± 87.8	0.086
NLR	90 min	3.92 ± 3.68	2.05 ± 3.77	0.003	149.1 ± 133.9	84.6 ± 104.4	<0.001
PLR	5 min	−14.22 ± 57.34	−9.31 ± 114.76	0.768	−0.8 ± 38.6	12.6 ± 67.0	0.177
PLR	45 min	−16.88 ± 113.39	−39.91 ± 166.11	0.354	−2.4 ± 81.7	5.3 ± 70.6	0.504
PLR	90 min	−8.21 ± 81.07	−39.54 ± 165.66	0.201	4.0 ± 47.9	7.6 ± 74.4	0.748
SII	5 min	−518.39 ± 509.81	−603.50 ± 369.38	0.182	−62.3 ± 22.8	−55.4 ± 18.4	0.025
SII	45 min	−149.65 ± 819.30	−117.30 ± 591.82	0.751	−4.3 ± 107.3	−1.8 ± 49.6	0.806
SII	90 min	278.81 ± 672.69	49.82 ± 662.31	0.039	59.6 ± 98.6	18.0 ± 56.7	<0.001
SIRI	5 min	−0.00 ± 1.23	0.98 ± 2.12	0.002	18.7 ± 75.3	81.4 ± 124.8	0.001
SIRI	45 min	2.24 ± 4.38	4.09 ± 4.68	0.013	183.6 ± 365.2	258.8 ± 288.5	0.122
SIRI	90 min	4.90 ± 4.16	4.67 ± 4.68	0.755	312.7 ± 270.7	295.7 ± 305.4	0.729

Absolute change was calculated as the cardiopulmonary bypass time point value minus the induction value. Percentage change was calculated as [(time-point value − induction value)/induction value] × 100. Analyses for the 90 min time-point were restricted to patients who remained on cardiopulmonary bypass for ≥90 min. Values are presented as mean ± standard deviation. Ex = mortality cases; Non-Ex = sampled surviving controls; NLR = neutrophil-to-lymphocyte ratio; PLR = platelet-to-lymphocyte ratio; SII = systemic immune-inflammation index; SIRI = systemic inflammatory response index; CPB = cardiopulmonary bypass.

**Table 5:** Univariate logistic regression for in-hospital mortality (clinical/operative).

Predictor	OR	95% CI	*p*
Age	1.062	1.028–1.097	**<0.001**
Male	1.058	0.556–2.016	0.863
DM	1.217	0.655–2.261	0.534
HT	0.767	0.412–1.430	0.404
EF	0.992	0.964–1.021	0.597
Pump time	1.043	1.030–1.055	**<0.001**
Cross-clamp time	1.115	1.081–1.150	**<0.001**

Statistical tests applied include univariate logistic regression analysis to evaluate the association between clinical and operative variables and in-hospital mortality. Odds ratios (OR) with corresponding 95% confidence intervals (CI) were calculated to quantify the strength and direction of the associations. Continuous variables were analyzed as continuous predictors, and categorical variables were analyzed as binary predictors. The statistical significance of each predictor was assessed using the Wald test. In the table, statistically significant values are marked in bold. The *p*-value indicates the level of statistical significance, where values less than 0.05 are considered statistically significant. OR = Odds Ratio, CI = Confidence Interval, EF = Ejection Fraction, DM = Diabetes Mellitus, HT = Hypertension.

**Table 6:** Univariate logistic regression for indices.

Index	OR	95% CI	*p*
NLR ind	1.134	0.989–1.300	0.073
PLR ind	1.007	1.004–1.010	**<0.001**
SII ind	1.001	1.000–1.001	**0.016**
SIRI ind	1.172	0.968–1.419	0.103
NLR 5	1.583	1.219–2.056	**<0.001**
PLR 5	1.010	1.006–1.014	**<0.001**
SII 5	1.004	1.003–1.006	**<0.001**
SIRI 5	1.742	1.408–2.155	**<0.001**
NLR 45	1.029	0.960–1.103	0.418
PLR 45	1.003	1.001–1.006	**0.006**
SII 45	1.002	1.001–1.003	**<0.001**
SIRI 45	1.283	1.103–1.492	**0.001**
NLR 90	1.074	0.954–1.209	0.238
PLR 90	1.002	0.999–1.004	0.158
SII 90	1.001	1.000–1.001	**0.009**
SIRI 90	1.163	1.020–1.327	**0.025**
NLR post	0.851	0.794–0.913	**<0.001**
PLR post	0.989	0.985–0.993	**<0.001**
SII post	0.999	0.999–1.000	**<0.001**
SIRI post	0.924	0.877–0.973	**0.003**

Statistical tests applied include univariate logistic regression analysis to evaluate the association between inflammatory indices measured at different during cardiopulmonary bypass and postoperative time points and in-hospital mortality. Odds ratios (OR) with corresponding 95% confidence intervals (CI) were calculated to assess the magnitude and direction of associations. Continuous variables were analyzed as continuous predictors. The statistical significance of each predictor was assessed using the Wald test. In the table, statistically significant values are marked in bold. The *p*-value indicates the level of statistical significance, where values less than 0.05 are considered statistically significant. Because PLR and SII have larger numerical scales than NLR and SIRI, their odds ratios should be interpreted per 1-unit increase with caution. To improve interpretability, rescaled analyses using 100-unit increments for PLR and SII were additionally considered, and the direction and statistical significance of the associations remained consistent. OR = Odds Ratio, CI = Confidence Interval, NLR = Neutrophil-to-Lymphocyte Ratio, PLR = Platelet-to-Lymphocyte Ratio, SII = Systemic Immune-Inflammation Index, SIRI = Systemic Inflammatory Response Index, ind = Baseline (Induction), 5 = 5th minute during cardiopulmonary bypass measurement, 45 = 45th minute during cardiopulmonary bypass measurement, 90 = 90th minute during cardiopulmonary bypass measurement, post = Postoperative measurement.

**Table 7:** Multivariable logistic regression models with discrimination, internal validation, and calibration performance for prediction of in-hospital mortality.

Model	Variable	OR	95% CI	*p*	AUC	Bootstrap-Corrected AUC	Optimism	Max VIF	ΔAUC vs. Model A	DeLong *p*	Calibration Intercept	Calibration Slope	Hosmer–Lemeshow *p*
Model A Clinical model	Age	1.064	1.030–1.099	**0.0002**	0.698	0.684	0.014	1.12	Reference	Reference	0.000	1.000	0.649
	EF	0.986	0.957–1.015	0.333									
	Pre-Creatinine	1.050	0.713–1.545	0.806									
Model B Primary: Clinical + NLR 5 min	Age	1.055	1.022–1.090	**0.0011**	0.729	0.713	0.016	1.34	0.031	0.143	0.000	1.000	0.573
	EF	0.984	0.955–1.014	0.306									
	Pre-Creatinine	1.105	0.742–1.645	0.623									
	NLR 5 min	1.480	1.128–1.940	**0.0046**									
Model C Primary: Clinical + SII 5 min	Age	1.061	1.026–1.096	**0.0005**	0.797	0.781	0.016	1.41	0.099	**0.0007**	0.000	1.000	0.792
	EF	0.986	0.955–1.017	0.364									
	Pre-Creatinine	1.143	0.754–1.732	0.528									
	SII 5 min	1.004	1.002–1.006	**<0.001**									
Model D Primary: Clinical + SIRI 5 min	Age	1.044	1.011–1.079	**0.0091**	0.787	0.771	0.016	1.29	0.089	**0.0055**	0.000	1.000	0.351
	EF	0.988	0.958–1.019	0.448									
	Pre-Creatinine	1.139	0.752–1.726	0.539									
	SIRI 5 min	1.625	1.303–2.026	**<0.001**									
Model E Secondary exploratory: Clinical + NLR 5 min + SII 5 min	Age	1.073	1.035–1.113	**0.0001**	0.821	0.804	0.017	4.62	0.123	**<0.001**	0.000	1.000	0.261
	EF	0.987	0.956–1.018	0.406									
	Pre-Creatinine	1.125	0.738–1.715	0.584									
	NLR 5 min	0.486	0.258–0.913	**0.025**									
	SII 5 min	1.008	1.004–1.012	**0.0001**									
Model F Secondary exploratory: Clinical + NLR 5 min + SIRI 5 min	Age	1.043	1.009–1.078	**0.013**	0.791	0.774	0.017	3.88	0.093	**0.0036**	0.000	1.000	0.225
	EF	0.990	0.960–1.022	0.546									
	Pre-Creatinine	1.114	0.733–1.694	0.612									
	NLR 5 min	0.683	0.407–1.146	0.148									
	SIRI 5 min	2.061	1.386–3.065	**0.0004**									
Model G Secondary exploratory: Clinical + SII 5 min + SIRI 5 min	Age	1.051	1.016–1.087	**0.0042**	0.803	0.787	0.016	3.94	0.105	**0.0018**	0.000	1.000	0.053
	EF	0.987	0.956–1.019	0.431									
	Pre-Creatinine	1.170	0.766–1.788	0.467									
	SII 5 min	1.003	1.001–1.005	**0.017**									
	SIRI 5 min	1.311	0.993–1.729	0.056									

Statistical tests applied include multivariable logistic regression analysis to identify independent predictors of in-hospital mortality. Odds ratios (ORs) with corresponding 95% confidence intervals (CIs) were calculated for each variable. Model A included age, ejection fraction, and preoperative creatinine. Models B–D represent primary models in which each inflammatory index was added separately to the clinical model. Models E–G represent secondary exploratory models including combinations of inflammatory indices. Model discrimination was evaluated using receiver operating characteristic curve analysis, and AUC values were calculated for each model. Internal validation was performed using bootstrap resampling with 1000 iterations, and optimism-corrected AUC values are reported. AUC differences were calculated relative to Model A, and correlated AUCs were compared using DeLong’s test. Calibration was assessed using calibration intercept, calibration slope, and the Hosmer–Lemeshow goodness-of-fit test. Multicollinearity was assessed using the variance inflation factor (VIF), and the maximum VIF value for each model is reported. Because the study used an enriched nested case–control design, discrimination, calibration, and incremental performance metrics should be interpreted as exploratory and should not be considered externally validated absolute risk estimates. OR = odds ratio; CI = confidence interval; AUC = area under the curve; VIF = variance inflation factor; EF = ejection fraction; NLR = neutrophil-to-lymphocyte ratio; SII = systemic immune-inflammation index; SIRI = systemic inflammatory response index.

**Table 8:** Sensitivity Analysis Including Cardiopulmonary Bypass Duration and Aortic Cross-Clamp Time.

Model	Included Variables	OR (Inflammatory Index)	*p*-Value	AUC
Clinical + NLR-5	Age, EF, Preop Creatinine, CPB time, Cross-clamp time, NLR-5	1.24	0.466	0.977
Clinical + SII-5	Age, EF, Preop Creatinine, CPB time, Cross-clamp time, SII-5	1.003	0.062	0.979
Clinical + SIRI-5	Age, EF, Preop Creatinine, CPB time, Cross-clamp time, SIRI-5	1.35	0.198	0.979

Clinical model included age, ejection fraction, preoperative creatinine, cardiopulmonary bypass duration, and aortic cross-clamp time. OR: odds ratio; AUC: area under the receiver operating characteristic curve.

**Table 9:** ROC Performance of Inflammatory Indices at All Time Points for Prediction of In-Hospital Mortality.

Marker	Time Point	AUC	95% CI	Cut-Off (Youden)	Sensitivity	Specificity
NLR	ind	0.592	0.510–0.674	2.416	0.941	0.356
PLR	ind	0.649	0.567–0.731	246.903	0.431	0.907
SII	ind	0.698	0.622–0.774	623.050	0.863	0.490
SIRI	ind	0.586	0.504–0.668	1.208	0.922	0.278
NLR	5 min	0.681	0.605–0.757	3.167	0.588	0.809
PLR	5 min	0.687	0.611–0.763	275.000	0.431	0.954
SII	5 min	0.790	0.720–0.860	235.888	0.980	0.567
SIRI	5 min	0.765	0.695–0.835	2.014	0.863	0.613
NLR	45 min	0.563	0.483–0.643	6.743	0.392	0.866
PLR	45 min	0.649	0.571–0.727	162.617	0.608	0.778
SII	45 min	0.737	0.665–0.809	574.037	0.824	0.670
SIRI	45 min	0.693	0.621–0.765	4.002	0.627	0.696
NLR	90 min	0.574	0.494–0.654	4.119	0.392	0.809
PLR	90 min	0.589	0.509–0.669	170.930	0.569	0.732
SII	90 min	0.515	0.435–0.595	594.837	0.961	0.263
SIRI	90 min	0.542	0.462–0.622	3.615	0.863	0.258
NLR	Postoperative	0.757	0.685–0.829	7.917	0.725	0.778
PLR	Postoperative	0.789	0.719–0.859	137.956	0.804	0.840
SII	Postoperative	0.711	0.639–0.783	2389.889	0.961	0.428
SIRI	Postoperative	0.628	0.548–0.708	14.653	0.922	0.325

Statistical tests applied include receiver operating characteristic (ROC) curve analysis to evaluate the predictive performance of inflammatory indices measured at different time points for in-hospital mortality. The area under the curve (AUC) was calculated to quantify the overall discriminative ability of each marker. Optimal cut-off values were determined using the Youden index, defined as the maximum value of sensitivity + specificity − 1. Sensitivity and specificity were calculated based on these optimal cut-off values. Higher AUC values indicate better discriminative performance. The *p*-value indicates the level of statistical significance, where values less than 0.05 are considered statistically significant. ROC = Receiver Operating Characteristic, AUC = Area Under the Curve, NLR = Neutrophil-to-Lymphocyte Ratio, PLR = Platelet-to-Lymphocyte Ratio, SII = Systemic Immune-Inflammation Index, SIRI = Systemic Inflammatory Response Index, ind = Baseline (Induction), 5 = 5th minute during cardiopulmonary bypass measurement, 45 = 45th minute during cardiopulmonary bypass measurement, 90 = 90th minute during cardiopulmonary bypass measurement, post = Postoperative measurement.

## Data Availability

The datasets generated and/or analyzed during the current study are available from the corresponding author upon reasonable request.

## Abbreviations

CPB	Cardiopulmonary bypass
NLR	Neutrophil-to-lymphocyte ratio
PLR	Platelet-to-lymphocyte ratio
SII	Systemic immune-inflammation index
SIRI	Systemic Inflammatory Response Index
ROC	Receiver operating characteristic
AUC	Area under the curve
CI	Confidence interval
OR	Odds ratio
EF	Ejection fraction
Ex	Mortality occurred
Non-Ex	Mortality did not occur
SD	Standard deviation
